# Children, Cell Phones, and Reading Comprehension

**DOI:** 10.3390/bs16081263

**Published:** 2026-07-23

**Authors:** Patricia M. Greenfield, Marola Hanna, Rocio Burgos-Calvillo, Laura Rhinehart

**Affiliations:** 1 Department of Psychology, University of California, Los Angeles, CA 90095, USA; marolah135712@g.ucla.edu (M.H.); rbcienfuegos@g.ucla.edu (R.B.-C.); 2 Departments of Education and Psychology, University of California, Los Angeles, CA 90095, USA; laura.rhinehart@ucla.edu

**Keywords:** children, cell phones, texting, distraction, reading comprehension, notifications

## Abstract

In the last several years, reading comprehension has declined during elementary school in the U.S. Are cell phones to blame? There is a dearth of evidence concerning the cell phone impact on reading comprehension at the elementary school level. Our primary question was whether acquisition of a phone would be negatively related to the reading comprehension performance of third- and sixth-grade children. More specifically, would notifications or the mere presence of a phone reduce the concentration required for an optimal reading comprehension performance? Among 106 children, we studied 55 children in a sixth-grade group and 51 children in a third-grade group. The sample was linguistically diverse: English was the home language for 42%, Arabic for 30%, and Spanish for 28%. The experimental procedure used a repeated-measures design: Participants who brought phones to school the day of the study took a grade-appropriate reading comprehension test from the DIBELS 8th edition with their phone next to them and a different test from the same battery without their phone nearby or in sight. Participants who did not have a phone with them also took two reading comprehension tests from the DIBELS 8th edition; they were the same tests taken by participants in the experimental portion of the study who had phones with them. All participants completed a short survey about phone ownership and use, along with demographic questions. There was no effect of the experimental conditions on reading comprehension: Whether or not a child had their phone in sight made no difference to their reading comprehension performance. Nor did reading comprehension differ for students with or without phones at school the day of the study. This absence of short-term effects contrasted with the presence of long-term relationships: Possessing a personal cell phone was significantly associated with lower reading comprehension scores across the whole sample. However, for participants from non-English-speaking homes who had their own phones, reading comprehension was significantly better the earlier a child began texting and had access to games on their phone. For children from English-speaking homes who had their own phones, neither early mobile game access nor earlier initiation of texting was associated with better reading comprehension.

## 1. Introduction

Test taking is an important task in educational settings. Recent technological tools appear to have an impact on this test-taking behavior, suggesting that cell phones interfere with the concentration required to perform well on academic tests. In this paper, we examine this association in a sample of third- and sixth-grade elementary school students. This examination is particularly relevant because children’s cell phone ownership is on the rise. Averaging phone ownership rates for 8- and 9-year-olds (i.e., third graders), we find that, in 2015, 13% of 8- and 9-year-olds in the U.S. had their own smartphones. By 2021, the percentage had grown to 31.5%. Averaging phone ownership rates for 11- and 12-year-olds (i.e., sixth graders), we find that the percentage owning smartphones in 2015 was 36.5%. In 2021, it was 54% (Rideout et al., 2022). Data in the present study were collected at the end of 2024. We therefore were able to assess cell phone ownership in 2024 for the third and sixth graders and whether cell phone ownership by young children was higher in our sample than in the national sample three years earlier (Research Question 1).

To study the issue of whether cell phones interfere with the concentration required to perform well on academic tests, we selected reading comprehension as our target behavior and examined whether acquisition of a phone is negatively related to reading comprehension performance (Research Question 2).

### 1.1. Theoretical Framework: Cognitive Load Theory

Cognitive load theory (Plass et al., 2010; Sweller, 2011) is related to learning and assumes that there are limits to one’s working memory (i.e., humans can only work with a few pieces of information at a time). According to this theory, distracting or irrelevant information can introduce “extraneous cognitive load,” which can overload working memory. Extraneous cognitive load makes it more difficult for students to learn and can negatively impact performance. Cell phones, through calls, texts, notifications, or mere presence, can introduce extraneous cognitive load. Taken together, cognitive load theory suggests that, because working memory is limited, distractions from cell phones can tax this system and overload students’ cognitive capacity, thereby limiting their learning and performance. Cognitive load provides a theoretical rationale for our hypothesis: that cell phone notifications would reduce reading comprehension performance.

### 1.2. Empirical Studies of Distraction by Notifications

Cognitive load theory has also provided a rationale for empirical studies testing the costs of notifications. Research suggests behavioral, physiological, and experiential costs. In one study, French university students completed a Stroop test while receiving smartphone-style notifications. “Notifications triggered a transient slowdown in cognitive processing lasting approximately 7 seconds.” (Fournier et al., 2025, p. 1). This effect was reflected in pupil dilation, a physiological marker of cognitive load. In another study, university students spent one week with all notifications silenced and one week with notifications maximized “by keeping notification alerts on and their phones within their reach/sight” (Kushlev et al., 2016, Abstract). Participants experienced significantly higher levels of inattention in the Interrupt condition than in the Do-Not-Interrupt condition (Kushlev et al., 2016).

Our extensive literature review revealed no studies of notification effects on cognition in elementary or middle school students. Therefore, our attempt in the present study appears to be the first. However, the unequivocal evidence for distracting effects in university students led us to frame our examination of distracting effects as a hypothesis rather than as a research question. Specifically, we hypothesized that phone notifications would reduce the reading comprehension performance of third-grade and sixth-grade students.

### 1.3. Empirical Studies of Distraction by Cell Phones: High School and College Students

In this section, we characterize general distraction rather than the specific distraction of notifications by examining experimental studies, teacher reports, and cell phone bans. For example, an experimental study with college students between the ages of 20 and 24 revealed that the mere presence on their desk of their own smartphone (turned off, screen-covered) reduced performance on a concentration and attention test in comparison with a group that placed their turned-off phone in another room (Skowronek et al., 2023). Despite one failure to replicate (Ruiz Pardo & Minda, 2022), most studies of college students are in line with Skowronek et al.’s basic findings (Tanil & Yong, 2020; Thornton et al., 2014; Ward et al., 2017).

The experience of high school teachers is in accord with the predominant experimental findings in college students. According to a Pew Research Center survey conducted in 2023, 72% of high school teachers perceived that students being distracted by their cell phones is a major problem in their classrooms. In contrast, only 6% of elementary school teachers noted it was a major problem (Hatfield, 2024). Despite the lack of saliency for teachers, this issue is especially relevant in elementary school settings where young children must use their budding self-regulation skills to inhibit distractions to pay attention in class and complete assignments.

The dearth of evidence concerning the impact of phone bans in elementary school and the importance of developing attentional skills for academic tasks in elementary school led to Research Question 3: Would the mere presence of a phone reduce the reading comprehension performance of third-grade and sixth-grade students?

### 1.4. Cell Phone Use and Reading Proficiency

In the last several years, reading comprehension scores have declined during elementary school in the U.S. (National Center for Education Statistics, 2024). One potential rationale for this decline, at least in part, is the rise of cell phone use. Due to this national decline, many U.S. schools are focused on ways to improve their students’ reading achievement. On the one hand, texting and reading on a cell phone can support decoding (i.e., word reading) and reading comprehension. On the other hand, children spend most of their time on their phones watching videos (Rideout et al., 2022). Therefore, cell phones might harm literacy development because children are spending less time reading. In Argentina, a survey of middle school students indicated that cell phone dependency was associated with weaker reading habits and more negative attitudes towards reading (Hilt, 2019). More specifically, time spent texting is associated with a reduction in reading time (Li et al., 2024).

Only two studies have focused on relationships between children’s cell phone use and their reading skills. Based on 12,000 participants between 9 and 13 years, Li and colleagues (2024) found that more texting time was consistently (over a two-year period) associated with less reading time, as well as consistently associated with weaker development of the basic skill of reading aloud letters and words. Mediation analysis indicated that texting time predicted a decline in reading time; less reading time, in turn, predicted lesser skill in reading aloud letters and words, as well as smaller brain volume in most reading-related regions. In contrast, Hofferth and Moon (2012) found that children ages 10–18 with access to a cell phone who spent more time sending text messages had better reading comprehension. If we integrate the findings from these two studies, it appears that texting benefits the higher order skill of reading comprehension but is a negative influence on basic reading mechanics at the letter and word level. More time spent talking on the phone also has a negative association with word reading (Hofferth & Moon, 2012).

More research on the impact of cell phone use on children’s reading is needed. For example, neither Li and colleagues nor Hofferth and Moon compared the reading skills of children with personal phone access to those without access, a gap our study design fills when it addresses our second research question: How does acquisition of a personal cell phone relate to reading comprehension performance?

Another gap in these two studies is the developmental gap. The statistics of phone ownership indicate that children have been receiving their own phones across a wide range of ages (Pew Research Center, 2025). With respect to reading comprehension, is there an optimal time to give children a phone? First phones do not always have texting or calling capabilities. Is there an optimal time to begin texting? Is there an optimal time to begin using the phone for voice communication? Our Research Question 4 addresses these issues.

### 1.5. Effects of Cell Phone Bans in Schools

In an effort to reduce distraction from cell phones in order to increase academic performance, many schools have banned cell phones. A meta-analysis examining the impact of phones on the academic achievement of K-12 and university students found that cell phones had a small, negative effect (Kates et al., 2018). Campbell et al.’s (2024) scoping review found mixed results of bans across the grades. However, these analyses did not differentiate among age groups between kindergarten and 12th grade despite obvious developmental differences. Another meta-analysis found that banning phones had a positive effect on social well-being (e.g., reduced bullying) but no significant effect on academic performance (Böttger & Zierer, 2024). Empirical research on the impact of cell phone bans on learning in schools suggests more mixed results than the experimental studies or high school teachers’ observations; but these studies have generally been done with adolescents and college students (Campbell et al., 2024).

Bans assume that children are bringing their phones to school. To what extent is this the case in the elementary grades? This is a basic question in light of the growing implementation of cell phone bans in schools at all levels. Our study design allowed us to address this question (Research Question 5).

How do cell phone bans affect elementary students’ academic achievement? So far, no study has carried out a quasi-experiment in which the academic performance of children who bring phones to school is compared to the performance of those who do not. This comparison is very relevant to assessing the potential of a school cell phone ban at the elementary school level. The quasi-experimental component of our research design allows us to compare reading comprehension between children who bring phones to school and those who do not (Research Question 6). The answer to this question can provide information that is relevant to the effectiveness of a cell phone ban with elementary school children.

### 1.6. The Role of Language and SES: Elementary School Children

Only two studies of learning or academic achievement and cell phone use in younger children have been reported. A survey of Latinx children between 7 and 11 years of age (mean of 9.5 years) from low-income, predominantly Spanish-speaking immigrant homes found no relationship between current phone ownership or age of phone acquisition and school grades (Sun et al., 2023). In contrast, a study of high-SES fourth graders in Australia demonstrated negative effects of increasing mobile phone use over time on concentration (in a Stroop task), response inhibition (a Go/NoGo task), and spatial learning (a maze task) (Bhatt et al., 2017). Given that English proficiency in Australia is related to higher SES (Blake et al., 2018), the contrasting results in the two studies could well be attributed to both SES and proficiency in the national language. Our study sample made it possible to assess the role of language because it included both children from English-speaking homes and children from homes where two other languages were spoken—Spanish or Arabic. In the present study, we pursue the role of language spoken at home as a factor in the effect of cell phones on reading comprehension (Research Question 7).

### 1.7. Hypothesis and Research Questions

In sum, we aim to fill these research gaps by exploring one hypothesis and seven research questions. Based on our review of the literature, only one issue in the current study’s examination of cell phone usage and comprehension had unequivocal prior research findings to support a hypothesis. All other issues were therefore posed as questions.

- Research Question 1. In the current study, what percentage of third and sixth graders have their own phones? Has cell phone ownership risen since 2021 in these age groups?
- Research Question 2. Is performance on a reading comprehension test related to whether or not a child has a personal cell phone?
- Hypothesis. Phone notifications will reduce the reading comprehension performance of third-grade and sixth-grade students.
- Research Question 3. Would the mere presence of a cell phone next to a child reduce their reading comprehension performance?
- Research Question 4. For students who have their own phone, is there an optimal time vis-à-vis reading comprehension performance to (a) get a first phone, (b) start using it for voice communication, and (c) start using it to text?
- Research Question 5. What percentage of third and sixth graders bring their phones to school?
- Research Question 6. Does reading comprehension differ for students who bring phones to school compared with those who do not?
- Research Question 7. Do any phone variables differ in their associations with reading comprehension for children from English-speaking and non-English-speaking homes?

## 2. Materials and Methods

### 2.1. Participants

In the original plan for our study, we aimed to solicit a sample of sixth graders based on evidence found in a national survey where about half of U.S. sixth graders had their own phones (Rideout et al., 2022). This goal would have allowed us to examine a sample at an ideal stage at which to compare the cognition of children with and without phones. However, in line with the more recent data, data from a pilot study for the current research suggested that many children received their own phones at a much younger age. In our pilot sample, the average age at which children first had a phone was seven. Children in our pilot study generally received a phone between kindergarten and third grade. So, for our full-scale study, reported here, we added a third-grade sample.

For the current study, our total sample included 106 children. This sample had both younger (*n* = 51) and older (*n* = 55) subgroups. Within the younger group, 47 were third graders. The younger subgroup also included two rising third graders, one child who had just completed third grade, as well as one child who was in fourth grade. Although not actively in third grade, these four children were added to the younger group. In the rest of this paper, we label this younger group “third graders”. Within the older group of participants, 43 were in the sixth grade. Five additional children, tested in the summer, had just completed sixth grade. Four children were currently in fifth grade; three were currently in seventh grade. These 12 children were added to the older group. In the Results Section, we label the older group “sixth graders”.

All participants resided in Los Angeles County, California. Drawing from a single region is the standard practice for in-person experiments with children. However, Los Angeles County is a geographically large area with multiple ethnically, linguistically, and socio-demographically distinct areas. Our locations covered four distinct geographical areas separated by approximately 78 miles north–south and 38 miles east–west. Ethnic and linguistic compositions also varied in our catchment areas.

Fifty-seven percent of the sample was female and 43% male. Forty-two percent of the sample was from English-speaking homes; 28% was from Spanish-speaking homes; and 30% was from Arabic-speaking homes. Children (58.5%) were assessed in a public school setting with 41.5% in a Coptic Christian church Sunday School setting. All students attended schools where instruction was provided in English. Seventy-one percent of the children who reported having their own phones were allowed to bring them to school.

Because of the political situation, with one of our groups extremely threatened by the deportation policies of the current federal government, we could not ask for demographic information on our survey. However, available evidence suggests SES differences are unlikely to explain the observed effects:

1. The design of the true experiment involved within-participant comparisons; therefore, each component comparison held SES constant.
2. *T*-tests indicated that the children from the two language groups—that is, those from English-speaking homes and those from Spanish- or Arabic-speaking homes—were not significantly different on any of the three reading comprehension measures—total errors (*p* = .45), reading scores (*p* = .52), or percentage correct (*p* = .61). In addition, a chi-square test indicated that children from English-speaking households, compared with children from Arabic- or Spanish-speaking households, did not have significantly different phone access (chi-square = 0.742, *p* = .39). These findings indicate that SES differences are not confounding the results.

For the experimental portion of our study, a power analysis indicated that to achieve 90% power within each age group (third grade/sixth grade) and experimental condition (phone/no phone), we would need 29 children in each grade. Given that the experimental condition (with or without phone) was a within-subject variable, this would mean 58 participants, separating the two age groups. However, our total N for the experimental portion of the study was 37. Therefore, we combined the two age groups, which provided an N of 37 in each within-subject condition. If phone use actually produces a decrement in cognitive performance, 90% power means that we would have a 90% chance of detecting that effect with 29 participants. Thus, 37 participants exceeded the minimum needed for 90% power. To control for developmental differences, we then used age group as a covariate. For the quasi-experiment, the 37 children who brought phones to school the day of the experiment were compared with the 69 children who did not bring phones to school that day.

### 2.2. Experimental Design and Procedure

Between July and December 2024, we collected data for an experiment in which each participant completed a reading comprehension test under two conditions: a repeated-measures design (1) with their phone turned on and face up next to their exam; (2) without their phone present. In the latter condition, participants gave their phone to one of the researchers before beginning the test.

Although one would expect reading comprehension to develop in the six months covered by our data collection, especially in third-grade students, level of reading comprehension was not a variable in this study. Comprehension was simply compared for each child with a phone present and a phone absent at the same sitting. The quasi-experiment also compared reading comprehension for children who brought a phone to school on the testing day with that of those who did not. Hence, date of testing was equated in the comparison, so results would not be affected by the fact that data collection took about six months. Neither are correlations affected by the long period of data collection because the correlated variables were always assessed on the same day.

Two maximally matched grade-appropriate reading comprehension tests were used, one for each condition. In terms of the research design, crossing two stories with two conditions (phone and no phone) yielded four counterbalanced orders for children who had their phones at school the day of testing. Children who had their phones with them at school were randomly assigned to one of the four conditions, making this portion of the study a true experiment.

The point of the four order conditions was to eliminate the effect of order by counterbalancing it; it was not to explore the effect of order on reading comprehension performance. Below are the orders with the number of children in each of the four conditions. As you can see, counterbalancing was not quite perfect: Conditions 1, 2, and 3 had ten children each, but Condition 4 had only seven children:

(1) Phone with Story 1; No Phone with Story 2 (*n*= 10);
(2) No Phone with Story 2; Phone with Story 1 (*n* = 10);
(3) Phone with Story 2; No Phone with Story 1 (*n* = 10);
(4) No Phone with Story 1; Phone with Story 2 (*n* = 7).

These 37 children were totaled as the experimental sample in order to align with our power analysis.

Because they could receive notifications, two children who were wearing smartwatches were included in the sample of 37 children who participated in the experimental portion of this study. Therefore, their smartwatches were taken away from them in the “no-phone” condition.

Children who did not yet have a phone or who did not bring their phone to school the day of testing also took the same two reading comprehension tests, with the order of the two stories counterbalanced. This group enabled a quasi-experimental design: we compared the reading comprehension performance of children who brought phones to school with that of those who did not.

A second quasi-experimental component of the study design related to assessing the effect of naturally occurring notifications. Notifications were also a quasi-experimental variable because they were not randomly assigned by the researchers. Before the phone condition, a researcher deleted any existing notifications from the children’s phones. After the phone condition, a researcher instructed the children not to erase any new notifications; the researcher then looked at each child’s phone and recorded the number of notifications received during the reading comprehension test (each story tested had a seven- minute time limit).

### 2.3. Assessing Reading Comprehension

**Figure 1:**
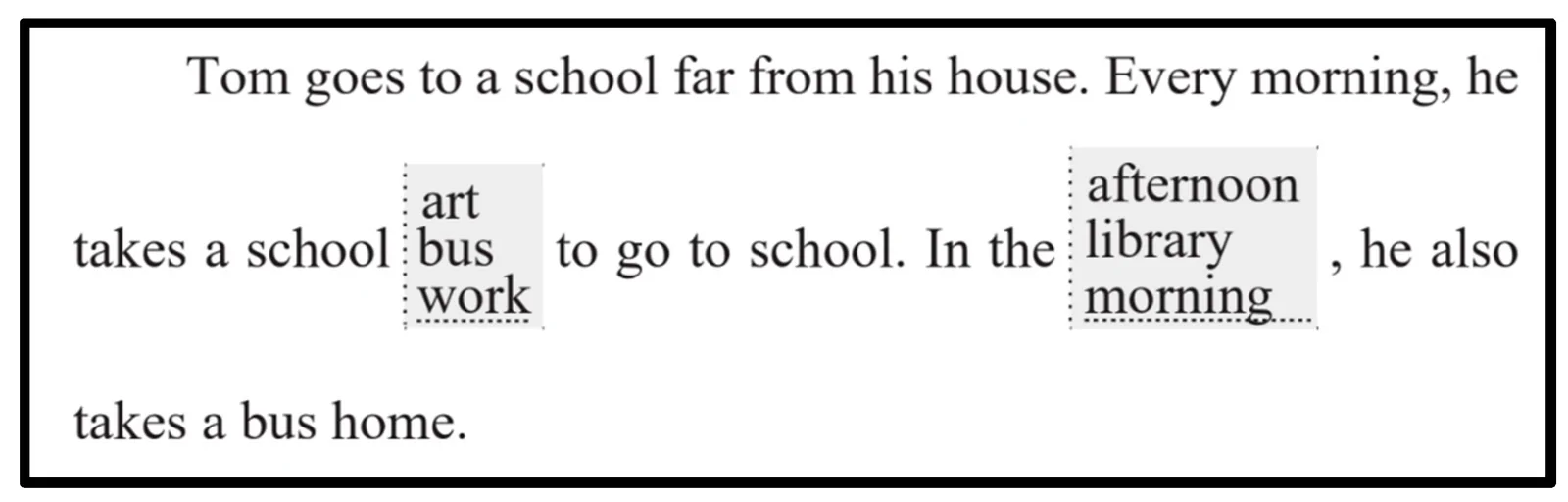
Practice Passage for Reading Comprehension Tests. Source: University of Oregon (2021).

For this purpose, we used the third-grade and sixth-grade Maze assessments from the 2018 Dynamic Indicators of Basic Early Literacy Skills (DIBELS) 8th edition from the University of Oregon. This is a multiple-choice test of skill in comprehending short stories. Figure 1 shows the practice passage used for both the third-grade and sixth-grade tests:

The test taker must select the correct word in each multiple-choice block. Scores are based on the number of correct choices for each story. The number of items is much greater on the two sixth-grade tests (64 items and 62 items per test) compared with that on the third-grade tests (48 items and 46 items); in addition, reading skills improve as children grow older and advance in school. We therefore controlled for grade in school whenever the whole sample, comprising both age groups, was analyzed together.

If any student struggled with reading or understanding the survey (the survey is described in the next section), the researchers provided help. If anyone struggled too much with the survey, they were asked if they could read in English. On the basis of this information, the few participants who could not read in English were eliminated from this study, and their test packets were taken away. Hence, these children are not included in the final sample of 106 children. All the children attended schools where instruction was provided in English. However, a few children in the Coptic churches had recently arrived in the U.S. from Egypt and were not yet accustomed to the language. Because of not being able to read English, these children were eliminated. The overwhelming majority of the children tested in the Coptic churches had been in the U.S. longer or learned English in Egypt. Therefore, they were able to take the exam with no literacy issues. Language spoken at home did not create literacy problems in our participant sample. This fact was shown by the virtually identical percentage-correct scores on the reading comprehension tests: a mean of 85% correct for children from English-speaking homes and a mean of 86% correct for children from Arabic- or Spanish-speaking homes.

### 2.4. Survey

Our survey collected background information on phone ownership, the age and grade at which the child first received a phone, their initial and current phone use (e.g., calling, texting, social media, gaming) and whether and when they were allowed to bring their phone to school. The complete survey is shown in Table 1.

A growing body of research indicates that elementary-aged children can provide accurate and reliable self-report data when questions are age-appropriate and concrete, which was the case with our instrument. For example, large-scale validation work on the Pediatric Quality of Life Inventory (PedsQL 4.0) demonstrates that children as young as five show minimal missing responses, internal consistency values above 0.70, and Total Scale Score reliabilities near or above 0.90. The measure also shows strong construct validity, with medium-to-large effect size differences between healthy children and those with chronic health conditions (Varni et al., 2007). More recent work provides additional support. Validation studies of the Very Short Well-Being Questionnaire for Children (VSWQ-C) and the developmentally adapted Definitional Positive and Negative Effect Schedule for Children (dPANAS-C) indicate that children as young as six can provide responses with acceptable reliability, strong convergent validity, and meaningful variation across demographic groups (Smees et al., 2020). Taken together, these findings indicate that age-appropriate instruments can yield valid and interpretable self-report data from children as young as six years of age. In our study, children were asked, for example, to report in what grade they first started texting. This is a clear and memorable event, so children are likely to remember it better than more abstract or emotional information. Although any kind of recall can have limitations, the research shows that it is reasonable to use self-reports from children in this age group.

Some of the first set of five participants left early without completing the survey when it was given after the reading comprehension tests. Therefore, they could not be used for any analyses that involved survey data. Their data were usable and were used for the experiment and quasi-experiment. However, we subsequently administered the survey before the reading comprehension tests to ensure that this background information was collected and completed. The final survey instrument is shown in Table 1.

Based on the data provided by the third- and sixth-grade samples to these demographic survey questions, our research questions and hypothesis needed to be considered with allowable data based on the children’s responses. Questions 2–11 in the survey (Table 1) required students to have their own phone. With a 96% response rate, 60 students reported having their own phone (22 third graders and 38 sixth graders); hence, these are the maximum samples for analyses involving Questions 2, 3, and 4, which relate to having your own phone. However, because Questions 5–11 in the survey instrument were added after the first 13 participants were tested, the maximum sample size for analyses involving Questions 5–11 is composed of participants starting with the 14th participant who also had their own phone. Forty-nine participants who were given Questions 5–11 reported having their own phones. Therefore, the maximum *n* for analyses involving Questions 5–11 was 49 participants. Analyses involving the grade of first calling also required students to have the current ability to call. Based on the survey responses, 30 sixth graders could currently call on their own phones; 11 third graders could currently call on their own phones. Hence 41 was the maximum *n* for analyses involving grade of first calling. Twenty-eight sixth graders and 11 third graders reported being able to text on their own phone; thus the maximum *n* for analyses involving grade of first texting was 39. Taking these factors into account, response rates are presented in the relevant results.

### 2.5. Research Questions, Hypothesis, and Analyses

The hypothesis and seven research questions were addressed by means of the following analyses:

#### 2.5.1. Research Question 1: What Percentage of Third and Sixth Graders Have Their Own Phones?

Responses to the survey question “Do you have your own phone?” supplied the answer to this descriptive question. Has cell phone ownership risen since 2021 in these age groups? Comparison with figures from Rideout et al. (2022) was used to answer this question.

#### 2.5.2. Research Question 2: Is Performance on a Reading Comprehension Test Related to Whether or Not a Child Has a Personal Cell Phone? 

This question was addressed by a correlation between cell phone ownership (yes or no) and continuous measures of reading comprehension for the sample as a whole, with grade partialled out. Adding performance on the two test forms together, the reading comprehension measures consisted of the total score, total errors, and percentage correct (total score divided by total attempted).

#### 2.5.3. Hypothesis: Phone Notifications Will Reduce the Reading Comprehension Performance of Third-Grade and Sixth-Grade Students 

To test this hypothesis, we ran a partial correlation (with grade as the control variable) between the number of notifications during the phone condition and the three reading comprehension measures (score, errors, percentage correct) during the phone condition.

#### 2.5.4. Research Question 3: Would the Mere Presence of a Cell Phone Next to a Child Reduce Their Reading Comprehension Performance? 

Each child who had a phone at school was tested on reading comprehension under two experimental conditions: participants read and responded to one test story with a phone turned on and face up next to them; participants read and responded to another test story with their phone temporarily taken away by the researcher. Stories were counterbalanced across conditions. To answer this research question, we carried out repeated-measures analyses of covariance (ANCOVA) with the experimental condition as the independent variable and grade as a covariate (to control for developmental differences). Three ANCOVAs were carried out using three different comprehension measures as the dependent variable: percentage correct (number of comprehension items answered correctly divided by number attempted), number of comprehension errors, and comprehension score (number of correctly answered items). All three dependent variables were assessed via a single reading passage in each experimental condition.

#### 2.5.5. Research Question 4: For Students Who Have Their Own Phone, Is There an Optimal Time to Get a Phone That Will Maximize Reading Comprehension? Once Children Have Their Own Phone, Is There an Optimal Time to Begin Texting or Using the Phone for Voice Communication? 

For children who reported having their own phone, (22 third graders and 38 sixth graders), grade of acquiring a personal cell phone was correlated with three measures of reading comprehension based on summing across the two tests: percentage correct, number of errors, and total score in each age group. For children who had their own phone and could currently use it to text, a correlation was run between grade of first texting and the three measures of reading comprehension. For children who had their own phone and could currently use it to call, a correlation was run between grade of first calling and the three measures of reading comprehension. For this research question, separate analyses were carried out for each grade level.

#### 2.5.6. Research Question 5: What Percentage of Third and Sixth Graders Bring Their Phones to School?

Because children had to have a phone at school to participate in the experimental portion of this study, the percentage of children in each grade participating in the experiment supplied the answer to this question.

#### 2.5.7. Research Question 6: Does Reading Comprehension Differ for Students Who Bring Phones to School Compared with That of Those Who Do Not? 

This was a quasi-experimental question because whether or not students had a phone at school was not a product of random assignment. To answer this question, we carried out an analysis of covariance with phone at school/no phone at school as the independent variable and grade as the covariate. Three ANCOVAs were carried out using each of the three different comprehension measures as the dependent variable: percentage correct (number of comprehension items answered correctly divided by number attempted), number of comprehension errors, and comprehension score (number of correctly answered items). For this analysis, each measure was created by summing across the two stories.

#### 2.5.8. Research Question 7: Do Any Phone Variables Differ in Their Associations for Children from English-Speaking and Non-English-Speaking Homes? 

Correlations of all phone variables with all comprehension variables addressed this question for each language background group (English-speaking homes, non-English-speaking homes). Grade was treated as a covariate for all correlations except texting. For texting, we limited our analysis to sixth graders because texting is more relevant to social life and reading level in sixth grade than in third grade.

All significance levels are based on two-tailed tests. For the correlational analyses, only the statistically significant results are reported.

### 2.6. Informed Consent

The study protocol was approved by the UCLA’s institutional review board (approval IRB#24-000365) on 24 June 2024. Assent was obtained from all participants and informed consent from their parents or legal guardians prior to participation in this study. Parent permission forms were provided in both English and Spanish, as appropriate, to ensure understanding and accessibility. All Arabic-speaking parents also spoke English fluently, so we did not need an Arabic translation. In addition, student assent, available in both English and Spanish, was obtained using an age-appropriate assent form that clearly explained the purpose of this study, the procedures, and the voluntary nature of participation. All participants were informed that they could withdraw from this study at any time without penalty.

## 3. Results

### 3.1. Research Question 1: What Percentage of Third and Sixth Graders Have Their Own Phones? Has Cell Phone Ownership Risen Since 2021 in These Age Groups?

In our third-grade sample, 46% (22/48) reported having their own phones. Among sixth graders, 70% (38/54) reported having their own phones (overall response rate: 96%). These rates of phone ownership are higher than the national rates reported in 2021; the rates three years earlier were 31.5% of third-graders and 54% of sixth graders (Rideout et al., 2022). For students in each grade who reported having their own phone, Table 2 shows when they reported receiving their first personal cell phone.

### 3.2. Research Question 2: Is Performance on a Reading Comprehension Test Related to Whether or Not a Child Has a Personal Cell Phone?

With grade controlled, correlations between having a cell phone and all three reading comprehension measures were conducted. We found a statistically significant relationship between having a personal cell phone and lower reading scores (*r* = −.223, *p* = .026, *df* = 97, 94% response rate). In terms of the differences between children from English-speaking and non-English speaking homes reported in Section 3.8, it is notable that this negative relationship between phone ownership and reading scores held across both language groups.

### 3.3. Hypothesis: Phone Notifications Will Reduce the Reading Comprehension Performance of Third-Grade and Sixth-Grade Students

Partial correlation (with grade level partialed out) indicated that notifications in the condition where students had their phones turned on next to them were not significantly related to any of the three measures of reading comprehension (response rate: 100%). The main reason for the lack of association of notifications with reading comprehension was undoubtedly that they were infrequent. Eight out of 10 third graders received zero notifications. Twenty out of 27 sixth graders received zero notifications.

### 3.4. Research Question 3: Would the Mere Presence of a Cell Phone Next to a Child Reduce Their Reading Comprehension Performance?

In the phone condition, children correctly answered 89% of the reading comprehension questions. In the no-phone condition, children correctly answered 87% of the reading comprehension questions. A repeated-measures analysis with phone condition vs. no-phone condition as the repeated measure, using grade level as a covariate, revealed that whether or not children had access to their phones did not make a significant difference in their percentage correct on the reading comprehension test. Neither did grade level make a significant difference. Details of this analysis are presented in Table 3. The same absence of a significant experimental effect occurred with the other two dependent variables, comprehension errors and comprehension scores.

### 3.5. Research Question 4: For Students Who Have Their Own Phones, Is There an Optimal Time to Get a Phone That Will Maximize Reading Comprehension? Once Children Have Their Own Phone, Is There an Optimal Time to Begin Texting or Using the Phone for Voice Communication?

For the 38 sixth graders (70% of the sixth-grade sample) who reported having their own phone, there were no significant correlations between the grade at which a child acquired their first phone and any measure of reading comprehension. The same pattern held for the 22 third graders (46% of the third-grade sample). The response rate was 95% in both age groups.

There was also no significant correlation for sixth graders who had their own phone and could currently text between grade of first texting and any measure of reading comprehension. The response rate was 96%. The same held for third graders; their response rate was 100%.

Nor was there a significant correlation in either age group for children who had their own phone and could currently call between grade of first calling and any measure of reading comprehension. The sixth-grade response rate was 97%; the third-grade response rate was 100%.

### 3.6. Research Question 5: What Percentage of Third and Sixth Graders Bring Phones to School?

Eighteen percent (*n* = 9) of the full sample of 51 third graders had a phone at school the day of our reading comprehension tests. Fifty-one percent (*n* = 28) of the full sample of 55 sixth graders had a phone with them at school on the day of testing.

### 3.7. Research Question 6: Does Reading Comprehension Differ for Students Who Bring Phones to School Compared with That of Those Who Do Not?

The answer to this question is “no.” Combining the two grade levels and using grade as a covariate, analysis of covariance showed no significant difference in reading comprehension (percentage correct) for the 36% of children who had phones with them the day of the experiment and the 64% who of children who did not (*N*_total_ = 102). Grade was a significant covariate. Third graders answered 79% of attempted comprehension questions correctly. Sixth graders answered 89% of attempted comprehension questions correctly. ANCOVA details are in Table 4 (96% response rate). The other two measures of reading comprehension yielded the same pattern of results.

### 3.8. Research Question 7: Do Any Phone Variables Differ in Their Associations for Children from English-Speaking and Non-English-Speaking Homes?

Separating sixth graders from English-speaking and non-English-speaking homes, we found different patterns in the two groups. For children from non-English-speaking homes, the *earlier* sixth graders with their own phones had started texting, the better their reading comprehension scores (*r* = −.576, *p* = .04, *n* = 13). For children from English-speaking homes, the trend went in the opposite direction, but the correlation was not statistically significant: the *later* sixth graders with their own phones had started texting, the better their reading comprehension scores (*r* = .26, *p* = .32, *n* = 17) (overall response rate: 79%).

There was a second difference between children from English-speaking and non-English-speaking homes. With grade controlled, there was a significant positive association for children with their own phones from non-English-speaking homes between playing games on their first phone and percentage of correct comprehension responses (*r* = .42, *p* = .03, *df* = 25), as well as an association between game playing on their first phone and fewer comprehension errors (*r* = −.53, *p* = .005, *df* = 25). The correlations for children from English-speaking homes were nonsignificant (overall response rate: 87%).

## 4. Discussion

Our study is unique in that it includes an experiment, took place in school environments populated by third- and sixth-grade students, and included a survey. This ecological validity enabled the researchers to answer a range of questions relating to children, cell phones, and reading comprehension—a key academic skill.

We did not find immediate effects of cell phone distraction in the experimental portion of our study. Given the data, this was not unexpected. As cognitive load theory suggests (Plass et al., 2010; Sweller, 2011), extraneous cognitive load, in the form of cell phone notifications, can impede learning and performance. However, our participants received very few notifications. Thus, it is possible that the notifications did not introduce extraneous cognitive load and did not overload students’ working memory.

However, because Cheever et al. (2014) found that for college students and their phones, “out of sight is not out of mind”, and other researchers have found a distracting effect of the mere presence of phones for college students (e.g., Skowronek et al., 2023), we had expected a distracting effect of the phone, even without notifications. This expectation was not confirmed.

We think that one reason that our results are not confirming our hypothesis is the interfering effect of anxiety about not having a functioning phone in sight when a researcher took the phone away. That is, students could have experienced nomophobia (anxiety over being separated from the phone) when their phones were taken away (Notara et al., 2021). Indeed, this effect has been found in college students and young adults (Cheever et al., 2014; Notara et al., 2021). We therefore think that anxiety over having one’s phone taken away may interfere with reading comprehension as much as distraction by the phone itself. That is, nomophobia might have artificially suppressed scores in the “no-phone” condition, masking a true distracting effect.

One practical implication might be that, as distracting as phones might be, outlawing phones in school might make students more anxious. Studies show a dose-related effect of screen use on both anxiety over separation from mobile devices (Cheever et al., 2014) and ADHD symptoms (Ra et al., 2018). However, there are two other potential solutions that produce neither anxiety nor distraction: One is a high level of engagement in activities such as sports or playing a musical instrument that preclude the use of digital devices (Uhls et al., 2014). The other is the use of educational curricula in school to help young people cope with the digital environment (e.g., R. Greenfield, 2024, 2025).

However, our quasi-experiment comparing the reading comprehension of students who had phones with them at school with that of those who did not was an analysis that overcame these limitations. The results nonetheless revealed the same pattern as the experimental results: the naturalistic presence of having a phone at school was not associated with any difference in reading comprehension compared with students who did not have a phone at school. This whole pattern of results indicates that cell phone bans at the elementary school level might not make a difference for learning or cognition.

Our results differed from our prior study of screens in a college classroom in which putting screens away in the classroom was associated with a better test performance (Rhinehart et al., 2021). However, that was a more long-term effect covering weeks within a college term. Indeed, in the present study, we did find a negative association between phone ownership and a measure of reading comprehension—again a long-term effect. Although this finding is correlational, the fact that phone ownership occurred temporally before the reading comprehension tests makes this a longitudinal finding, one prerequisite for establishing causality.

There is of course the possibility of selection effects, that is, that struggling students are given phones rather than that phone ownership causes lower reading comprehension scores. Two facts, one internal to the study and one external, make this an extremely unlikely, if not impossible, causal chain. The internal finding is that 59% of the 102 children who responded reported owning their own phone. It is unlikely that more than half the sample would be having difficulties in school. The external reason is that to improve or compensate for poor academic functioning is not among the top five reasons why parents give their children their own phone (Fernandez et al., 2025). Hence, consideration of the facts surrounding children’s phone ownership buttresses the tentative conclusion that this association between phone ownership and lower reading comprehension is an important long-term negative effect of children’s phone ownership.

For children who have their own phones, a long-term but unexpected effect was the positive association between starting to text earlier and reading comprehension for sixth graders who came from Arabic- or Spanish-speaking homes. For children from non-English speaking homes who are given phones, texting might provide important practice in reading English, compensating for the negatives of phone ownership in the domain of reading comprehension. Similarly, for children from non-English-speaking homes who were given phones, being able to game on their first phone was associated with better reading comprehension. Studies have shown that informal, out-of-school exposure can substantially support children’s English language development, with interactive and multimodal practices such as gaming, social media, and speaking linked to significant proficiency gains (De Wilde et al., 2020).

### Limitations and Future Directions

The scarcity of notifications was the major limitation of the experimental portion of this study. One reason there were few notifications was that each reading comprehension test was only seven minutes long. Indeed, the disruptive effect of cognitive disruption in French university students was greater when the average number of notifications received per day was greater and checking behavior was more frequent (Fournier et al., 2025). Extrapolating from the French university students to the U.S. children in our study, a low rate of notifications would minimize cognitive disruption of reading comprehension. Fournier et al. (2025) also found that notification frequency, but not screentime, was the factor that produced a decrement in focused attention. If this finding replicates with children, turning notifications off could be a more effective and easier-to-employ educational and parental strategy for maximizing children’s cognitive and academic performance than either phone bans or screentime limitations. This conclusion is buttressed by a study of university students in the United States (Kushlev et al., 2016). Two hundred twenty-one students agreed “to minimize phone interruptions by keeping alerts off and their phones away. Participants reported higher levels of inattention and hyperactivity when alerts were on than when alerts were off.” The authors conclude that “These findings highlight some of the costs of ubiquitous connectivity and suggest how people can reduce these costs simply by adjusting existing phone settings” (Kushlev et al., 2016, Abstract).

We treated smartwatches as potential distractors, just like smartphones. We therefore took them away during the “no-phone” condition of the experiment. Although smartwatches were a minority phenomenon (2/37), it is probably better to think of the experiment as testing the effect of distraction by notifications in general rather than as a test of distraction by a particular device, the smartphone.

Because teens use phones socially, text frequently, frequently check and multitask with their phones, and often receive a plethora of notifications (L. Greenfield, 2024; Toh et al., 2019), researchers would be more likely to find significant negative effects of notifications on the concentration needed for cognitive and academic tasks in a teenage group. We therefore have turned our research attention to high school students. However, rather than replicate this experimental procedure with adolescents, we are currently assessing the needs for and effects of a digital curriculum (Hanna et al., 2026).

When told about the absence of experimental effects in our study, a 19-year-old digital native remarked, “Nobody looks at their phone when they are taking a test.” So that might be another reason why cell phone presence did not disturb reading comprehension. Hence, using a test to assess academic accomplishment is also a limitation of this study. This limitation can be corrected in future experimental research by using an academic or cognitive task that is not structured as a test.

Furthermore, reading comprehension was the sole academic outcome measured in the present study. Therefore, notifications or ownership might exert stronger effects on other cognitive or academic domains, such as sustained attention, working memory, writing, or mathematical problem solving. Thus, the generalizability of the findings to broader academic functioning is limited. Moreover, although this study assessed phone ownership and age of initiation of texting and gaming, it did not capture the qualitative nature of phone use (e.g., types of games, language of texting, educational vs. entertainment content, or social versus solitary use). These distinctions might be particularly important, given the differential associations observed for children from English- versus non-English-speaking homes, and their absence limits interpretation of the mechanisms underlying the long-term relationships.

Although this study revealed associations between earlier access to mobile phones and later reading comprehension, these findings were based on data collected at a single time point. Longitudinal research is needed to establish whether early phone use affects subsequent reading comprehension development, whether children with stronger reading skills are more likely to engage in particular phone-related activities, and whether both outcomes are influenced by unmeasured third variables.

Another limitation was the fact that we did not measure nomophobia. Therefore, we were unable to assess the extent to which anxiety about having one’s phone taken away counteracted the distracting effect of cell phone presence. In other words, nomophobia possibly functioned as an unplanned and unmeasured neutralizer of the experimental effect.

A final limitation was the small sample size for comparing children from Englishspeaking and non-English-speaking homes in each age group. This situation most likely led to the statistical nonsignificance of the finding that the *later* sixth graders who had their own phones and were from English-speaking homes had started texting, the better their reading comprehension scores.

## 5. Conclusions

Our quasi-experimental analysis comparing the reading comprehension of students who had phones with them at school with that of those who did not was not subject to the limitations of the experimental portion of this study. The results nonetheless indicated the same pattern: the naturalistic presence of having a phone at school was not associated with any decrement in reading comprehension compared with children who did not have a phone with them at school. In short, indications of short-term effects of cell phone use and ownership were lacking. In contrast, there was strong evidence for long-term relationships. We therefore hypothesize that distraction may be a cumulative or habit-forming effect as much as an immediate one. Most important, we found a negative long-term association between cell phone ownership and reading comprehension that held across the entire sample. The implication is that parents concerned about reading skills should avoid giving children their own cell phones during elementary school.

Other long-term effects were specific to children from non-English-speaking homes. For children from non-English-speaking homes who possessed their own phones, starting to text in a lower grade was associated with better reading comprehension. For these children, texting might be useful in learning the host country language, which of course was used in our reading comprehension tests. For these same children, using their first phone to play games was associated with better reading comprehension. This gaming experience may be giving children who do not have access to other electronic devices an opportunity to develop both print and digital literacy (e.g., P. M. Greenfield, 1984/2014).

Because of the absence of experimental or quasi-experimental effects, our results do not provide an academic rationale for banning phones in elementary school. Studies of bans in other countries have also found mixed cognitive/learning effects (see Campbell et al., 2024 for a comprehensive review).

It is more likely that cell phone bans in elementary school will address and correct social rather than cognitive problems. Our earlier experiment with college students comparing in-person communication with a friend to text, phone, and video calls with the same friend indicated a significantly greater feeling of closeness after in-person communication (Sherman et al., 2013). Dwyer et al. (2018) found that, for college students and adult community members, smartphone use undermined enjoyment of face-to-face social interaction. These effects could potentially occur in elementary school, where social relations and social skills with peers are first developed. Because cell phone bans in school will force greater in-person communication, and this in-person communication will occur without the presence of phones, close social relations and social skills should benefit.

## Figures and Tables

**Table 1:** Survey: Demographics and history of cell phone ownership and use.

Girl or boy? (Circle one)	Grade:	Age:	Date:
School:	Language spoken at home:		
1. Do you have your own phone?			
2. If yes, how old were you when you first got your own phone?			
3. If you have your own phone, what grade were you in when you first received it?			
4. If you have your own phone, circle the things you were allowed to do when you first got your own phone: Call Text Social media (e.g., Instagram, Facebook, Snapchat, Tiktok) Play games			
5. How old were you when you first used your phone for calling?			
6. What grade were you in when you first used your phone for calling?			
7. How old were you when you first used your phone for texting?			
8. What grade were you in when you first used your phone for texting?			
9. Are you allowed to bring your phone to school?			
10. If so, what grade were you in when you first were allowed to bring your phone to school?			
11. What can you do now with your phone? Call Text Social media (e.g., Instagram, Facebook, Snapchat, Tiktok) Play games			

**Table 2:** Grade when personal cell phone was first acquired.

		Third-Grade Students		Sixth-Grade Students	
		*n*	%	*n*	%
Reported Grade of First Phone	TK	3	14.3	0	0
	KG	1	4.8	0	0
	First grade	4	19.0	1	2.8
	Second grade	13	61.9	7	19.4
	Third grade	0	0	7	19.4
	Fourth grade	0	0	11	30.6
	Fifth grade	0	0	4	11.1
	Sixth grade	0	0	5	13.9
	Seventh grade	0	0	1	2.8

Note: TK—transitional kindergarten; KG—kindergarten. One of the 22 third graders and 2 of the 38 sixth graders who reported having a phone did not note when they received their first phone. Hence, this table shows a total third-grade *n* of 21 and a total sixth-grade *n* of 36.

**Table 3:** Effect of phone access on comprehension.

	Independent Variable				Repeated-Measures ANCOVA		Covariate	
	With Phone		Without Phone		Phone vs. No Phone		Grade: Third or Sixth	
Reading Comprehension: Percentage Correct	*M*	*SD*	*M*	*SD*	*F*(1, 35)	*p*	*F*(1, 35)	*p*
% Correct out of Number Attempted	89%	16%	87%	14%	0.18	.678	2.11	.155

**Table 4:** Effect of bringing phone to school on reading comprehension.

	Independent Variable				ANCOVA	Covariate
	Phone at School (*n* = 37)		No Phone at School (*n* = 65)		Phone vs. No Phone at School	Grade: Third or Sixth
Comprehension Variable	*M*	*SD*	*M*	*SD*	*F*(1, 99)	*F*(1, 99)
% Correct out of Number Attempted	88%	15%	83%	16%	0.30	8.78 *p <* .01

## Data Availability

Data will be available on the OSR site.
